# Mikania Micrantha Wilt Virus Alters Insect Vector’s Host Preference to Enhance Its Own Spread

**DOI:** 10.3390/v11040336

**Published:** 2019-04-09

**Authors:** Rui-Long Wang, Keyan Zhu-Salzman, Mohammed Esmail Abdalla Elzaki, Qiao-Qiao Huang, Shi Chen, Zhi-Hui Ma, Shi-Wei Liu, Jia-En Zhang

**Affiliations:** 1 Key Laboratory of Agro-Environment in the Tropics, Ministry of Agriculture, College of Natural Resources and Environment, South China Agricultural University, Guangzhou 510642, China; rlw2009@scau.edu.cn (R.-L.W.); 13924082629@163.com (Z.-H.M.); 13711379414@163.com (S.-W.L.); 2 Departments of Entomology, Texas A&M University, College Station, TX 77843, USA; ksalzman@tamu.edu; 3 College of Crop Science, Fujian Agriculture and Forestry University, Fuzhou 350000, China; alzaki_mohammed@yahoo.com; 4 Environment and Plant Protection Institute, Chinese Academy of Tropical Agricultural Sciences, Haikou 571101, China; cosplete@126.com; 5 College of Materials and Energy, South China Agricultural University, Guangzhou 510642, China; chenshi@scau.edu.cn

**Keywords:** Mikania micrantha wilt virus, *Myzus persicae*, virus-induced plant volatile, virus-host plant-insect vector interactions, odor cues, *Gentian mosaic virus*, Fabavirus

## Abstract

As an invasive weed, *Mikania micrantha* Kunth has caused serious damage to natural forest ecosystems in South China in recent years. Mikania micrantha wilt virus (MMWV), an isolate of the *Gentian mosaic virus* (GeMV), is transmitted by *Myzus persicae* (Sulzer) in a non-persistent manner and can effectively inhibit the growth of *M. micrantha*. To explore the MMWV-*M. micrantha*-*M. persicae* interaction and its impact on the invasion of *M. micrantha*, volatile compounds (VOCs) emitted from healthy, mock-inoculated, and MMWV-infected plants were collected, and effects on host preference of the apterous and alate aphids were assessed with Y-shaped olfactometers. Gas chromatography-mass spectrometry (GC-MS) analysis indicated that MMWV infection changed the VOC profiles, rendering plants more attractive to aphids. Clip-cages were used to document the population growth rate of *M. persicae* fed on healthy, mock-inoculated, or MMWV-infected plants. Compared to those reared on healthy plants, the population growth of *M. persicae* drastically decreased on the MMWV-infected plants. Plant host choice tests based on visual and contact cues were also conducted using alate *M. persicae*. Interestingly, the initial attractiveness of MMWV-infected plants diminished, and more alate *M. persicae* moved to healthy plants. Taken together, MMWV appeared to be able to manipulate its plant host to first attract insect vectors to infected plants but then repel viruliferous vectors to promote its own dispersal. Its potential application for invasive weed management is discussed.

## 1. Introduction

*Mikania micrantha* Kunth (Asteraceae), commonly known as mile-a-minute weed, is a perennial vine. Although native to Central and South America, it has now become an important invasive weed in the Southeast Asia and Pacific regions [1]. Among the 10 worst weeds in the world, *M. micrantha* is a serious threat to the functioning local ecosystems and global biodiversity [1,2,3,4]. Due to its rapid growth, wide ecophysiological tolerance, strong reproduction, high phenotypic plasticity, and allelopathic inhibitory effect on neighboring plants [1,5], *M. micrantha* invasion in Southern China has caused substantial damage to natural ecosystems [1,2,5]. To limit the invasive weed, mechanical removal, chemical, biological, and ecological controls have been implemented over the past few years [3]. However, no single method can effectively reduce the losses caused by *M. micrantha* [1,2,3].

The majority of plant-infecting viruses are transmitted to their host plants by vectors [6]. Modes of insect-transmitted plant viruses are categorized into four types: (a) Non-persistently transmitted, stylet-borne; (b) semi-persistently transmitted, foregut-borne; (c) persistently transmitted, circulative; and (d) persistently transmitted, propagative [7]. Non-persistently transmitted (e.g., *Cucumber mosaic virus* (CMV)) and semi-persistently transmitted (e.g., *Tomato chlorosis virus* (ToCV)) viruses do not breach the gut barrier in their vectors; rather they are retained in the insect stylet or foregut prior to transmission [8]. Such viruses can be transmitted within seconds to hours after acquisition [8]. Persistent transmission, however, requires days to weeks [6,7]. Persistently transmitted circulative viruses (e.g., *Potato leafroll virus* (PLRV)) are able to cross the gut barriers and circulate in the insect hemolymph, but do not replicate in the insect vector. Persistently transmitted propagative viruses (e.g., *Southern rice black streaked dwarf virus* (SRBSDV) circulate and replicate in the insect vector [8]. Non-persistently transmitted viruses are acquired via short epidermal probes, which occur on the host and non-host plants of the vector during host selection, whereas persistently-transmitted viruses require phloem ingestion to be transmitted, which needs a specific vector of a suitable host-plant [6,7,8].

It is known that insect-transmitted plant viruses have developed different strategies to manipulate host-vector interactions and that the transmission mechanism greatly influences the strategies [9]. Persistently transmitted viruses tend to improve the host quality and promote long-term feeding, leading to higher reproductive rate, crowding, accelerated use of host resources, and eventual dispersal of viruliferous vectors [9,10,11]. Non-persistently transmitted viruses, on the other hand, often reduce plant quality, which inevitably increases vector restlessness and opportunities for virus acquisition and promotes rapid dispersal [9,10,11]. Infection of *Camelina sativa* by *Turnip yellows virus* (TuYV, circulative-persistent, non-propagative) enhanced palatability for *Myzus persicae* (Sulzer) [12]. The intrinsic growth rate of *M. persicae* was significantly higher and the pre-reproductive period was significantly shorter on plants infected by TuYV compared to uninfected plants [12]. Similarly, the pre-reproductive period of *Bemisia tabaci* females fed on tobacco plants infected with *Tomato yellow leaf curl China virus* (TYLCCNV, transmitted in a circular, persistent manner) was drastically improved, being 7-fold higher in longevity and 18-fold higher in fecundity than those on non-infected plants [13]. *Aphis gossypii* (Glover) and *M. persicae* grew more slowly on CMV-infected cucumber plants than those on uninfected plants indicative of reduced host quality [14].

Plant viruses, regardless of their mode of transmission, influence host-derived cues which tend to enhance vector attraction to infected hosts [9]. For example, *A. gossypii* and *M. persicae* preferred CMV-infected cucumber plants to healthy and mocked-inoculated plants [14]. More aphids were found to be attracted to tobacco plants infected with the *Tobacco etch virus* (TEV, transmitted in a non-persistent manner) than to healthy plants in a field evaluation experiment [15]. Virus-infected plants emit volatiles to makes them more attractive to insect vectors, which obviously would promote virus acquisition. However, after virus acquisition, the relative preference of the vector may shift to uninfected plants [14,16,17,18]. *M. persicae* reared on healthy *Physalis floridana* Rydb plants preferred to settle on potato plants infected by PLRV, whereas viruliferous *M. persicae* reared on PLRV-infected *P. floridana* plants preferred to settle on mock-inoculated potato plants [19]. Similarly, non-viruliferous *Rhopalosiphum padi* (L.) aphids preferred wheat infected by *Barley yellow dwarf virus* (BYDV), but once the aphids have acquired this virus, their settling preferences change, favoring healthy plants [16]. This subsequent host preference changes most likely would facilitate virus transmission and spreading after the vectors become viruliferous [14,16,17,18,20].

Mikania micrantha wilt virus (MMWV), an isolate of *Gentian mosaic virus* (GeMV) in the genus *Fabavirus* of the *Secoviridae* family, was initially found in 2008 in Guangdong Province, China. *M. persicae* can efficiently transmit this virus in a non-persistent manner [2,4,21]. MMWV-infected *M. micrantha* displays wilt, leaf crimpling and malformation symptoms [2]. Because of the inhibitory effects on host plant growth and reproduction, MMWV has been proposed to be used as a control agent for *M. micrantha* [2,4].

Our previous field observations and laboratory experiments found that more *M. persicae* initially settles on MMWV-infected *M. micrantha* leaves [2,4]. To investigate whether MMWV-*M. micrantha*-*M. persicae* interaction enhances transmission of MMWV, we analyzed the plant volatile organic compounds (VOCs) emission before and after MMWV infection. We documented *M. persicae* orientation preference in response to volatile cues originating from healthy, mock-inoculated or MMWV-infected-plants. Aphid population growth was also measured on healthy, mock-inoculated, and MMWV-infected plants. In addition, the initial and after-host-contact distributions of alate *M. persicae* were analyzed on healthy, mock-inoculated, and MMWV-infected plants.

## 2. Materials and Methods

### 2.1. Insects

The *M. persicae* colony was originated from field-grown rapeseed (*Capsicum annuum* L.) at Research Farm Field, South China Agriculture University, Guangzhou (N 23°16′, E 113°34′) and maintained on soybean (*Glycine max* (L.) Merr.) in an insectary (25 ± 2 °C, 70 ± 5% relative humidity and a 14 h light: 10 h dark photoperiod) for more than eight months.

### 2.2. Plants

*M. micrantha* plants at vegetative stage approximately 150 cm tall were collected in May 2017 from the same Research Farm Field as described above. They were cut into 10 cm long pieces and planted in plastic pots (8.5 cm diameter; 7 cm height) in a greenhouse (25 ± 1 °C, 75 ± 5% relative humidity, 14 h light: 10 h dark photoperiod). Plants were allowed to climb on 70 cm bamboo sticks and watered with diluted Hoagland solution (25% *v/v*) twice a week.

### 2.3. MMWV Inoculation Procedures

MMWV, also obtained from the same location in May 2016, was maintained on *M. micrantha* in a glasshouse at South China Agriculture University, Guangzhou. Young, highly symptomatic leaves of MMWV-infected *M. micrantha* were ground in 0.5 M potassium phosphate buffer (pH 7.5). Plants used for infection were mechanically inoculated with MMWV. To inoculate, the fourth and fifth leaves (counted basipetally from the apex) of the plants (about 45 cm tall) were dusted with carborundum powder and inocula were spread over the leaves using a cotton-tipped applicator [14]. Mock-inoculated plants were generated by applying inoculation buffer to the leaves at the same positions.

### 2.4. Choice Tests

For choice tests based on olfactory cues, a Y-shaped tube (2 cm inner diameter) consisting of a central tube (13 cm long) and two arms (10 cm long, offset by 60°) was connected to 36 L glass desiccators. Five healthy, mock-inoculated, or MMWV-infected plants (10 days after inoculation) were placed in glass desiccators, respectively. The GC-grade air was pumped into the glass desiccators at a flow rate of 200 mL min^−1^. The Y-tube was housed in an airtight cubical box (70 cm × 45 cm × 30 cm) with a 25 W filament lamp giving a constant light 25 cm above each arm. The temperature in the glass desiccators was 25 °C and the relative humidity was 70%. An *M. persicae* aphid (starved for 1 h before tests) was individually placed in the central tube and allowed to choose between the two arms. A choice was registered if *M. persicae* passed the threshold line located 3 cm away from the Y-junction. A “no choice” was marked if it failed to decide during the 5 min testing period [22]. Pairings for host preference tests were designed as follows: (i) Healthy vs. MMWV-infected, (ii) healthy vs mock-inoculated, and (iii) mock-inoculated vs. MMWV-infected. Thirty *M. persicae* were tested for each pairing. When 5 *M. persicae* individuals had been tested, the odor sources were interchanged. The Y-tube and plants were replaced after testing of 10 aphids. All Y-tubes were cleaned with 95% alcohol and dried in a 120 °C oven before use [20,22]. A total of 150 aphids in 5 replicates

Choice tests based on visual and contact cues followed the procedure of previous studies with minor modifications [14,16,23]. A glass Petri dish (12 cm diameter) lined with a filter paper was divided into two parts by pencil marks. Two leaves, each still attached to a differently treated plant, (i) healthy vs. MMWV-infected, (ii) healthy vs mock-inoculated, and (iii) mock-inoculated vs. MMWV-infected, were placed in the Petri dish, into which 50 alate *M. persicae* were released. Petri dishes were sealed with Parafilm perforated to allow air movement. The number of aphids settled on each leaf was counted 30 min and 60 min later, respectively. A total of 750 aphids in 15 replicates were tested for each dual-choice bioassay.

### 2.5. Collection of Plant Volatiles

**Figure 1:**
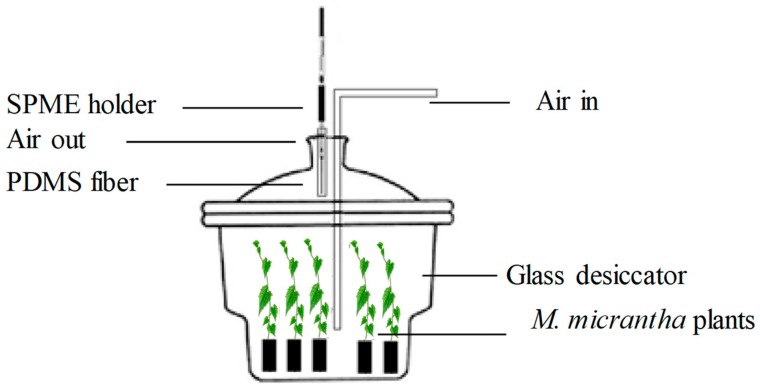
The schematic device used for the SPME extraction of volatile compounds (VOCs) emitted by *M. micrantha* plants.

Ten healthy, mock-inoculated or MMWV-infected plants (10 days after inoculation) were placed in a glass desiccator (36 L). Plants were illuminated with fluorescent bulbs (50 μmol m^−2^ s^−1^ with a photoperiod of 16 h light: 8 h dark). The temperature in the glass desiccators was 25 °C, and the relative humidity was 70%. The Pots with potting soil were wrapped with aluminum foil to prevent water evaporation and volatile emission from the soil. A watered pot wrapped in aluminum foil without plants was used as a negative control. The glass desiccator was connected to a GC-grade air generator (QL-3, Shandong Saikesaisi Hydrogen Energy Co., Ltd., Shandong, China) through a cork plug with two openings allowing gases to go in and out at a flow rate of 350 ml min^−1^. A clean Pasteur glass pipette was inserted in the outlet of the cock plug and VOCs were collected with divinyl-benzene/carboxen/polydimethylsiloxane (DVB/CAR/PDMS, 50 μm/30 μm, No.57348-U) solid-phase microextraction (SPME) fibers (Bellefonte, PA, USA). The manual SPME holder was purchased from Supelco (Bellefonte, PA, USA) (Figure 1). SPME fibers were conditioned at 250 °C for 1 h, and then placed in the Pasteur glass pipette to absorb VOCs for 2 h. All assays were performed in triplicate.

### 2.6. Volatile Analysis

The SPME fibers were removed and VOCs were analyzed using gas chromatography-mass spectrometry (GC-MS, TSQ Quantum XLS, Thermo Fisher Scientific Inc., USA) equipped with a TG-5HT column (30 m × 0.25mm × 0.25 μm, Thermo Fisher Scientific Inc., USA), helium (1 mL min^−1^ gas flow rate), a split injection temperature of 220 °C, a source temperature of 200 °C, an ionization potential of 70 eV, and a scan range of 35–450 amu. Samples were injected with an injection temperature of 220 °C, the oven temperature, starting at 60 °C (kept for 3 min), was increased (10°C min^−1^) to 120 °C, then further increased to reach 180 °C (5 °C min^−1^) and finally to 250 °C (25 °C min^−1^, and was kept for 3 min). Individual compound peaks were identified by reference library search using authentic standards and the National Institute of Standards and Technology MS database (NIST MS Search 2.0). Compounds were quantified on the basis of individual peak areas and the quantitative analysis of each volatile compound (expressed as a percentage of area) was determined by the peak area normalization method.

### 2.7. Population Growth of M. persicae

Five *M. persicae* apterous adults were placed in a clip cage (4 cm diameter × 3.5 cm height) with soft nylon mesh (size 80 mesh) covering one side to allow ventilation. The cage was clipped to a leaf of a plant (healthy, mock-inoculated or MMWV-infected) supported by a bamboo stick. All aphids except three neonate nymphs (foundresses) were removed 12 h later. The foundresses were checked daily and the total numbers of aphids produced was recorded after 10 days. The population growth rate was calculated as the increase in the number of aphids per colony per day per plant [24]. Fifty single-aphid replicates per treatment were included in the study.

### 2.8. Data Analysis

All statistical analyses were performed using SPSS 20.0 software (IBM, SPSS, Chicago, IL, USA). The distribution of alate *M. persicae* based on visual and contact cues of healthy, mock-inoculated, or MMWV-infected plants at 30 min and 60 min time points was analyzed using the generalized linear model. The population growth of *M. persicae* was analyzed by one-way ANOVA followed by Duncan’s multiple range test. Statistical differences were considered significant at *p* < 0.05. Principal Component Analysis (PCA) was used to analyze the relative peak area of each compound of the VOCs collected from healthy, mock-inoculated, or MMWV-infected plants.

## 3. Results

### 3.1. Odor from MMWV-Infected Plants is More Attractive to Aphids

**Figure 2:**
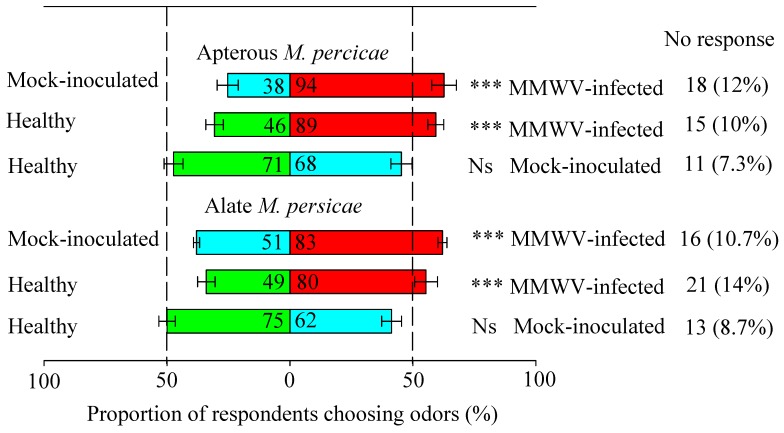
The response of *Myzus persicae* to different odor stimuli. For each pair of stimuli, a total 150 *M. persicae* were tested by the Y-shaped olfactometer. Data were presented as the mean ± SE. Bars denoted by asterisk indicate a significant preference for the treatment (* *p* < 0.05, ** *p* < 0.01, *** *p* < 0.001). Ns: No significant difference (*p* > 0.05). Healthy plants are shown in green; MMWV-infected plants are shown in red; Mock-inoculated plants are shown in blue. Numbers of individuals that responded or did not respond are given.

To determine the potential of volatiles in host preference of *M. persicae*, healthy, mock-inoculated, or MMWV-infected plants were evaluated in choice tests using a Y-tube olfactometer. Apterous *M. persicae* preferred the infected (59.3%) over healthy plants (30.7%) (*t* = 5.963, *df* = 8, *p* < 0.001) (Figure 2), although 10% did not make their choice. Likewise, the majority of aphids preferred the infected plants (62.7%) to the mock-inoculated plants (25.3%) (*t* = 5.671, *df* = 8, *p* < 0.001). No significant difference was found when choice was made between healthy and mock-inoculated plants (*t* = 0.346, *df* = 8, *p* = 0.738).

Similar observations were obtained with alate *M. persicae*: 53.3% aphids preferred the infected plants which were significantly more than that on healthy plants (32.7%) (*t* = 9.347, *df* = 8, *p* < 0.001). When facing a choice between mock-inoculated and MMWV-infected plants, 34.0% aphids selected the former and 55.3% preferred the latter (*t* = 3.635, *df* = 8, *p* < 0.001). No significant difference in aphid numbers was found between healthy and mock-inoculated plants (*t* = 1.658, *df* = 8, *p* = 0.136). These results indicated that odor from MMWV-infected plants was significantly more attractive to *M. persicae* than that of the healthy or mock-inoculated plants.

### 3.2. Aphid Distribution in Preference for Contact Cues

**Figure 3:**
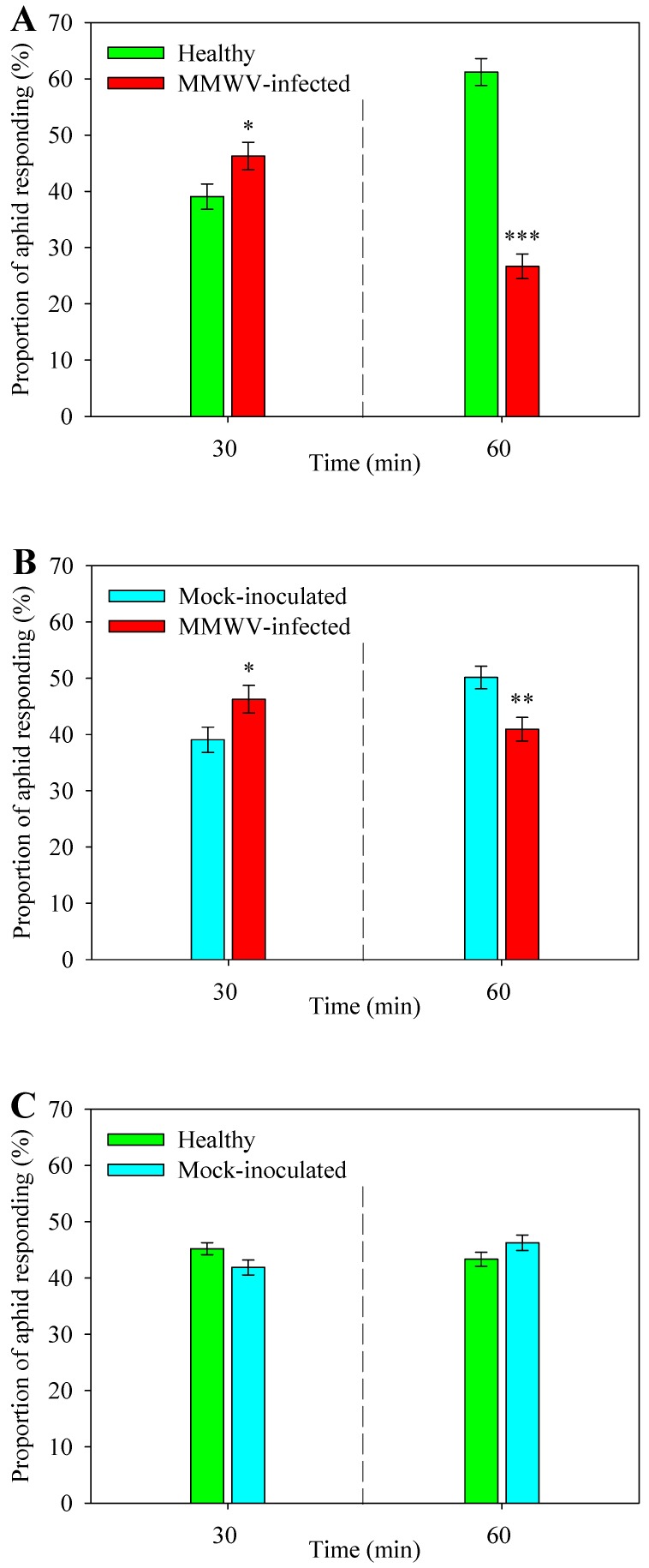
Distribution of alate *Myzus persicae* in response to contact cues of healthy, mock-inoculated and MMWV-infected plants after 30 min and 60 min. (**A**) Healthy vs. MMWV-infected plants, (**B**) Mock-inoculated vs. MMWV-infected plants, (**C**) Healthy vs. mock-inoculated plants. Each replicate (n = 15) consisted of fifty alate *M. persicae*. Asterisks indicate significant differences (* *p* < 0.05, ** *p* < 0.01, *** *p* < 0.001).

We also assessed the host preference of alate *M. persicae* based on visual and contact cues from plant leaves (Figure 3). At 30 min after release, 46.3% aphids *M. persicae* (*df* = 1, *F* = 4.697, *p* < 0.05) were found on MMWV-infected plants and 39.1% on healthy plants (Figure 3A). Also, 14.6% of *M. persicae* made no choice. Similarly, 48.1% were found on infected plants relative to 42.7% on mock-inoculated plants (*df* = 1, *F* = 6.122, *p* < 0.05) (Figure 3B). However, at the 60 min time point, for the infected vs. healthy plant pair, aphids on the MMWV-infected plants dropped to 26.7% (*df* = 1, *F* = 35.317, *p* < 0.001) compared to an increase to 61.2% on the healthy plants (*df* = 1, *F* = 45.493, *p* < 0.001) (Figure 3A). Likewise, on the infected vs. mock-inoculated plant pair, more aphids preferred mock-inoculated (50.1%) to MMWV-infected plants (40.9%) (*df* = 1, *F* = 10.038, *p* < 0.05) after 60 min (Figure 3B). In contrast, no significant difference was found between aphid numbers when choices were made between healthy and mock-inoculated plants at both 30 min (*df* = 1, *F* = 3.824 *p* = 0.061) and 60 min (*df* = 1, *F* = 2.523, *p* = 0.123) time points (Figure 3C). These results indicated that, although MMWV-infected plants were more attractive initially, more *M. persicae* emigrated to healthy plants once they probed or fed on infected plants.

### 3.3. Chemical Analysis of Volatiles

**Figure 4:**
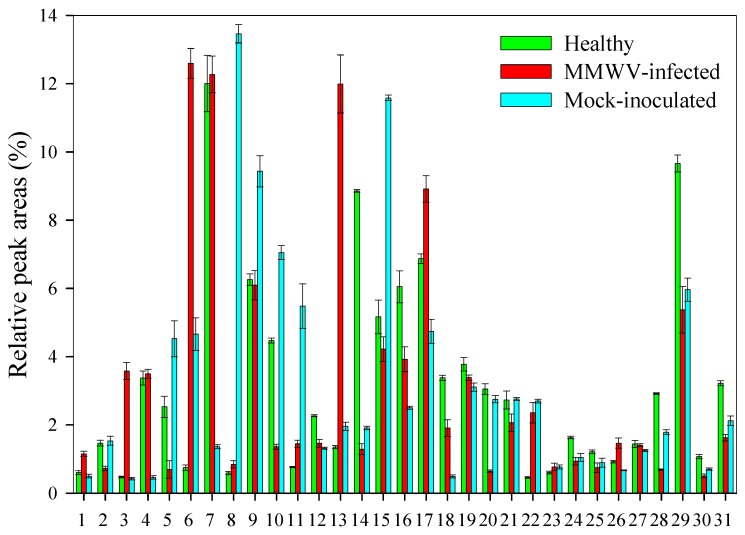
VOCs released from healthy, Mikania micrantha wilt virus (MMWV)-infected, or mock-inoculated plants. Quantitative analyses of each volatiles organic compound (expressed as area percentage) were carried out by a peak area normalization measurement. Values are mean ± SE (three replicates). 1, Hexanal; 2, 2-Hexenal; 3, α-Ocimene; 4, *α*-Cubebene; 5, *α*-Copaene; 6, *α*-Curcumene; 7, *β*-Cubebene; 8, Isocaryophillene; 9, *β*-Caryophyllene; 10, trans-*α*-Bergamotene; 11, Cedrene; 12, cis-*β*-Farnesene; 13, Humulene; 14, *β*-Himachalene; 15, Isoledene; 16, (+)-Epi-bicyclosesquiphellandrene; 17, α-Zingiberene; 18, *α*-Muurolene; 19, *β*-Bisabolene; 20, *β*-Sesquiphellandrene; 21, δ-Cadinene; 22, *β*-Vatirenene; 23, *α*-Guaiene; 24, Cyclohexene; 25, Isolongifolene; 26, (*E*)-Nerolidol; 27, ç-Himachalene; 28, ç-Muurolene; 29, Germacrene D; 30, trans-Sesquisabinene hydrate; 31, 8-oxo-9H-Cycloisolongifolen.

**Figure 5:**
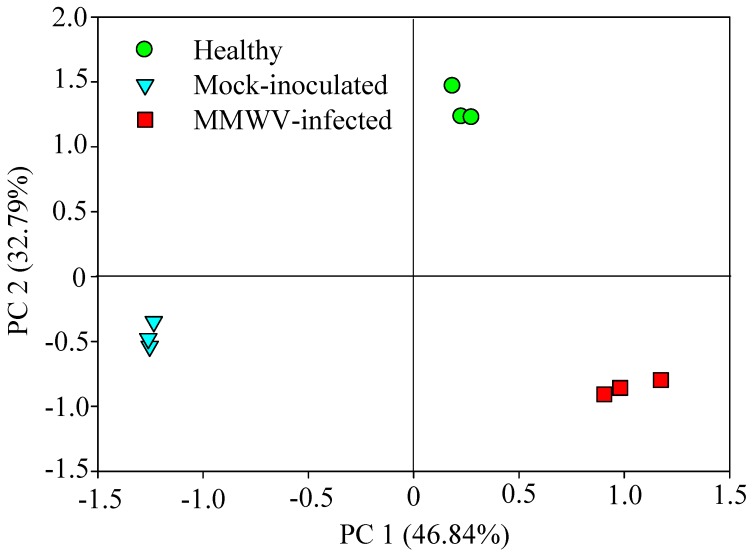
Principal component analysis (PCA) of VOCs released from healthy, MMWV-infected and mock-inoculated plants.

The volatiles from healthy, mock-inoculated, and MMWV-infested plants (10 days after inoculation) were analyzed by GC-MS (Figure 4). In total, 31 compounds were identified, of which, *β*-cubebene (12.0%), germacrene D (9.7%), *β*-himachalene (8.9%), α-zingiberene (6.9%), and *β*-caryophyllene (6.3%) were the main VOCs released from the healthy plants (Figure 4). In contrast, α-curcumene (12.6%), *β*-cubebene (12.3%), α-zingiberene (8.9%), and *β*-caryophyllene (6.1%) were the main compounds from MMWV-infected plants (Figure 4). Compared to the healthy plants, MMWV-infected plants significantly increased their emission of α-curcumene (94.1%), humulene (88.7%), and α-ocimene (86.7%), but decreased the emission of *β*-himachalene (586.4%), *β*-sesquiphellandrene (371.7%), and ç-muurolene (321.7%), respectively (Figure 4). Interestingly, isocaryophillene (13.5%), isoledene (11.6%), and *β*-caryophyllene (9.4%), trans-α-bergamotene (7.0%), and α-muurolene (6.0%) were found to be the major VOCs from mock-inoculated plants, distinct from those identified from healthy plants (Figure 4). On the other hand, no VOCs were detected from the negative control (pot wrapped in aluminum foil without plants). Perhaps plant handling, i.e., applying buffer to plants, could have introduced slight damage to the plants which was sufficient to cause changes in VOCs production. Such discrepancy has also been reported by other researchers [14,25]. GC-MS analysis revealed a significant increase in quantities of volatiles released by MMWV-infected plants relative to the healthy and mock-inoculated plants, but no qualitative differences were detected (Figure 4). The PCA analysis separated healthy, mock-inoculated, and MMWV-infected plants from each other (Figure 5, PC1 = 46.84%, PC2 = 32.79%). Therefore, MMWV-infection and plant handling significantly changed the VOCs profile of *M. micrantha*.

### 3.4. Effect of MMWV Infection on the Development of the M. persicae Population

**Figure 6:**
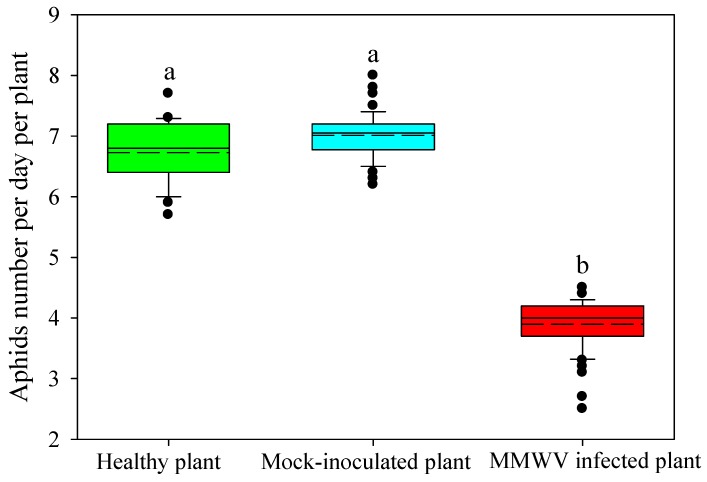
Effect of healthy plants (n = 50), mock-inoculated plants (n = 50), and MMWV-infected plants (n = 50) on the population growth rate of *Myzus persicae*. Box plot description: The long dashed line is the mean, the solid line is the median, the borders of the box are the first and third quartiles, the whiskers (error bars) represent 1.5 times the interquartile range, and points beyond the end of the whiskers are outliers. Different letters above bars indicate significant differences (*p* < 0.05) according to Duncan’s multiple range test.

We also examined the effect of host plants on aphid population growth and reproduction using clip-cages. (Figure 6). The average aphid growth rate on MMWV-infected plants was 3.90 aphids per day per plant, which was significantly lower than that on healthy (6.73 aphids per day per plant) and mock-inoculated plants (7.01 aphids per day per plant) (Figure 6). The 72.8% decrease indicated that MMWV-infested plants were not a suitable host for aphids compared to healthy plants.

## 4. Discussion

Plant viruses affect host plants in different ways to mediate vector attraction to, arrestment on, and dispersal from infected plants [9,16,19,25,26,27]. For example, aphids are attracted to plants infected with the non-persistent CMV and quickly left the plant after landing and avoided prolonged feeding. Such behavior presumably would enhance virus transmission particularly effectively with non-persistent viruses [14,25]. Persistently-transmitted viruses often promote long-term feeding as shown in *Sitobion avenae*, which is engaged in faster phloem-finding behavior and longer feeding time on plants infected with BYDV or PLRV than on non-infected plants [25,28]. A similar phenomenon has been observed with *R. padi*. Initially, non-infected *R. padi* significantly preferred wheat plants infected with BYDV compared to mock-inoculated plants, but ultimately viruliferous *R. padi* significantly preferred mock-inoculated wheat to virus-infected plants [16], which also would promote the spread of this persistently transmitted virus. In our MMWV-*M. micrantha*-*M. persicae* system, a change of plant host preference was also observed, supporting the “Host Manipulation Hypothesis” [14]. Gustatory, olfactory, and visual cues may have contributed to this preference switch and promotion of MMWV transmission.

Modified VOCs emissions due to virus infection can potentially serve as important mediators in decision making by insect vectors when selecting a host plant [25,29]. CMV-infected *C. pepo* in the field released significantly greater quantities of volatiles than healthy plants [14], presumably being responsible for attracting aphids to CMV-infected plants [14]. The total concentration of headspace VOCs differed between PLRV-infected and mock-inoculated potato plants as well. The concentration of monoterpenes, green leaf volatiles, and sesquiterpenes from PLRV-infected potato plants were higher than those of mock-inoculated plants [19]. Mock-inoculated tomato plants emitted significantly higher amounts of the terpenes *α*-pinene, 4-carene, *α*-phellandrene, terpinene, and *β*-phellandrene than *Tomato severe rugose virus* (ToSRV)-infected plants, indicating that ToSRV infection suppresses some volatile terpenes [25]. Pea plants inoculated with persistently transmitted viruses (e.g., *Bean leaf roll virus* (BLRV) or *Pea enation mosaic virus* (PEMV)) had significantly higher concentrations of green leaf volatiles relative to concentrations of monoterpenes, compared to either sham-inoculated or control plants [30]. In our study, *M. persicae* apparently could discriminate between healthy, MMWV-infected, or mock-inoculated plants possibly based on characteristics of the VOCs blends.

Virus-host plant-insect vector interactions could affect population growth of the insect vectors which would further influence virus transmission [16,31,32,33]. While some studies indicate that virus-infected plants are superior hosts for aphid reproduction [31,34,35], others show the opposite outcome [14,34]. In most cases, non-persistently transmitted viruses have negative effects on vector survival, fecundity or longevity, while persistently and semi-persistently transmitted viruses positively impact on one or more of these parameters [9]. For example, TYLCV mediated alterations to plant nutritional traits and defensive responses improve the growth and reproduction of *B. tabaci* Mediterranean (MED) [31]. MED feeding on TYLCV-infected plants gained 68% more weight and laid 81% more eggs than those feeding on mock-inoculated tomato plants. MED can both rapidly acquire and effectively transmit the persistently transmitted TYCLV. The beneficial effects of TYLCV infection on MED fitness should in turn favor TYLCV transmission [31]. Compared with non-infected sweet potato (*Ipomoea batatas*) plants, *M. persicae* displayed greater reproduction on the plants which were coinfected with the non-persistent transmitted viruses (*Sweet potato feathery mottle virus* (SPFMV), *Sweet potato virus G*, and *Sweet potato virus 2*) [34]. On the other hand, populations of *A. gossypii* and *M. persicae* were significantly reduced on the CMV-infected cucumber plants compared to those on healthy or mock-inoculated plants [14]. Also, compared to aphids on non-infected *Ipomoea hederacea* plants, the intrinsic rate of increase of *M. persicae* was significantly lower on SPFMV-infected plants, possibly due to an altered quality of infected plants [34]. In our study, the reduced population growth rate of *M. persicae* reared on the MMWV-infected plants indicates that MMWV-infected *M. micrantha* plants were also less suitable hosts. Under such a scenario, the negative effect of virus-infected plants may prompt vectors to depart and search for healthy, better-suited plants after landing and probing virus-infected plants. Dispersal of vectors would lead to the enhanced spread of MMWV.

Invasions of exotic species have become one of the major threats to ecosystem functioning and global biodiversity [35]. Some viruses have been suggested to be used to control of the invasive species [4,36,37,38,39,40,41,42,43]. For example, *Solenopsis invicta* virus 3 (SINV-3), *Tobacco mild green mosaic virus* (TMGMV) have been suggested to as a control agent for invasive species *Solenopsis invicta* [38,40] and *Impatiens glandulifera* [37,41], respectively. Although MMWV is not lethal for *M. micrantha*, it is the only virus that has been found to inhibit the growth and reproduction of *M. micrantha* [2,4]. Of course, extensive studies are needed to understand its natural reservoirs, host range, persistence in natural conditions, and the potential effects on crops and other plant species before considering MMWV as a candidate biocontrol tool for *M. micrantha*. Such detailed investigation is particularly important for non-persistently-transmitted viruses (e.g., MMWV) that can be transmitted by a potential vector landing on non-host plants and then starting its host selection behavior.

Taken together, this study provides more insight into virus-host plant-insect vector interactions. Future research should focus on the biological, chemical, or physiological processes and molecular mechanisms underlying MMWV-*M. micrantha-M. persicae* interactions.
